# Pathway analysis of bladder cancer genome-wide association study identifies novel pathways involved in bladder cancer development

**DOI:** 10.18632/genesandcancer.113

**Published:** 2016-07

**Authors:** Meng Chen, Nathaniel Rothman, Yuanqing Ye, Jian Gu, Paul A. Scheet, Maosheng Huang, David W. Chang, Colin P. Dinney, Debra T. Silverman, Jonine D. Figueroa, Stephen J. Chanock, Xifeng Wu

**Affiliations:** ^1^ Department of Epidemiology, The University of Texas M. D. Anderson Cancer Center, Houston, TX, USA; ^2^ Division of Cancer Epidemiology and Genetics, National Cancer Institute, Bethesda, MD, USA; ^3^ Department of Urology, The University of Texas M. D. Anderson Cancer Center, Houston, TX, USA

**Keywords:** pathway analysis, gene set enrichment analysis, GWAS, bladder cancer, susceptibility loci

## Abstract

Genome-wide association studies (GWAS) are designed to identify individual regions associated with cancer risk, but only explain a small fraction of the inherited variability. Alternative approach analyzing genetic variants within biological pathways has been proposed to discover networks of susceptibility genes with additional effects. The gene set enrichment analysis (GSEA) may complement and expand traditional GWAS analysis to identify novel genes and pathways associated with bladder cancer risk. We selected three GSEA methods: Gen-Gen, Aligator, and the SNP Ratio Test to evaluate cellular signaling pathways involved in bladder cancer susceptibility in a Texas GWAS population. The candidate genetic polymorphisms from the significant pathway selected by GSEA were validated in an independent NCI GWAS. We identified 18 novel pathways (*P* < 0.05) significantly associated with bladder cancer risk. Five of the most promising pathways (*P* ≤ 0.001 in any of the three GSEA methods) among the 18 pathways included two cell cycle pathways and neural cell adhesion molecule (NCAM), platelet-derived growth factor (PDGF), and unfolded protein response pathways. We validated the candidate polymorphisms in the NCI GWAS and found variants of *RAPGEF1, SKP1, HERPUD1, CACNB2, CACNA1C, CACNA1S, COL4A2, SRC*, and *CACNA1C* were associated with bladder cancer risk. Two *CCNE1* variants, rs8102137 and rs997669, from cell cycle pathways showed the strongest associations; the *CCNE1* signal at 19q12 has already been reported in previous GWAS. These findings offer additional etiologic insights highlighting the specific genes and pathways associated with bladder cancer development. GSEA may be a complementary tool to GWAS to identify additional loci of cancer susceptibility.

## INTRODUCTION

Urinary bladder cancer is the fourth most common cancer in men in U.S. Estimates in 2015 indicate urinary bladder cancer affects 56,320 males and 17,680 females, and will lead to 16,000 deaths in the U.S. [1]. Bladder cancer is a heterogeneous disease attributed to many risk factors. The number one risk factor is tobacco smoking, which explains 30-50% of bladder cancer risk [2]. Occupational exposure to chemicals [3, 4], genetic factors, and other environmental factors such as dietary factors, lifestyle factors, medical factors, fluid intake, also contribute to bladder cancer carcinogenesis [5], although some of the risk factors are inconclusive and vary in different studies. There is substantial evidence that there is an important genetic contribution to susceptibility to bladder cancer, initially based on familial clustering of bladder cancer. Epidemiologic studies have demonstrated a two-fold elevation in bladder cancer risk among first-degree relatives of bladder cancer patients [6, 7]. A segregation analysis in 1,193 families suggests a paucity of high penetrance gene for sporadic bladder cancer but instead many low penetrance genes with modest effects [8], indicative of a complex, polygenic model [9]. A large population based twin study evaluated the contribution of hereditary factors to the causation of various sporadic cancers and estimated the genetic contribution of bladder cancer to be roughly 30% [10].

Approaches towards mapping cancer susceptibility regions have undergone an evolution due to the recent annotation of variation across the genome as well as technical advances in single nucleotide polymorphism (SNP) arrays [11]. The candidate gene approach was pursued in the beginning but with very few successes that replicated in subsequent studies. In recent years, the emergence of genome-wide association studies (GWAS) has substantially advanced the field of identifying novel cancer susceptibility loci. Currently, bladder cancer GWAS have identified nine novel loci including 18q12.3 (urea transport, *SLC14A1*, intron), 8q24.21 (oncogene *MYC*, intergenic region), 4p16.3 (fibroblast growth factor, *FGFR3*, intron), 22q13.1 (apolipoprotein B mRNA-editing enzyme catalytic polypeptide-like 3A, *APOBEC3A*, intergenic region), 19q12 (cyclin E1, *CCNE1*, intergenic region), 8q24.3 (prostate stem cell antigen, *PSCA*, missense mutation), 3q28 (tumor protein p63, *TP63*, intergenic region), 2q37.1(UDP-glycosyltransferase 1 family polypeptide A1, *UGT1A*, intron), and 5p15.33 (telomerase reverse transcriptase, *TERT*, intron) [12–18]. In addition, two previously reported bladder cancer risk loci 1p13.3 (glutathiones transferase, *GSTM1*, deletion) and 8p22 (N-acetyltransferase 2, *NAT2*, intergenic region) were further validated in the GWAS studies [17, 19, 20].

Recent GWAS have provided valuable insights into the genetic basis of human disease but GWAS do not fully explain heritability in sporadic cancers [21]. So far, the low estimated effect sizes of the individual SNPs account for a small portion of the heritability of bladder cancer. In addition, majority of disease associated SNPs found in GWAS are tagging SNPs at non-genic regions without clear functional implication (http://www.genome.gov/gwastudies/). They may be highly correlated with the variants directly associated with bladder cancer susceptibility [22].

Gene set enrichment analysis (GSEA), also known as pathway based analysis, examines whether the test statistics for a group of common genetic variants that map to predefined gene sets (e.g., gene set from pathways defined by prior biological knowledge) support the possibility of disease association [23]. To date, GSEA have been applied to explore the critical pathways and genes of several diseases and traits, including Crohn's disease [24], rheumatoid arthritis [25, 26], multiple sclerosis [25, 27], diabetes [25], Parkinson's disease [25, 28, 29], bipolar disorder [25, 30], coronary artery disease [25], hypertension [25], age-related eye disease [25, 28], adult heights [31], colon cancer [32], breast cancer [33], and bladder cancer [34].

## RESULTS

**Figure 1:**
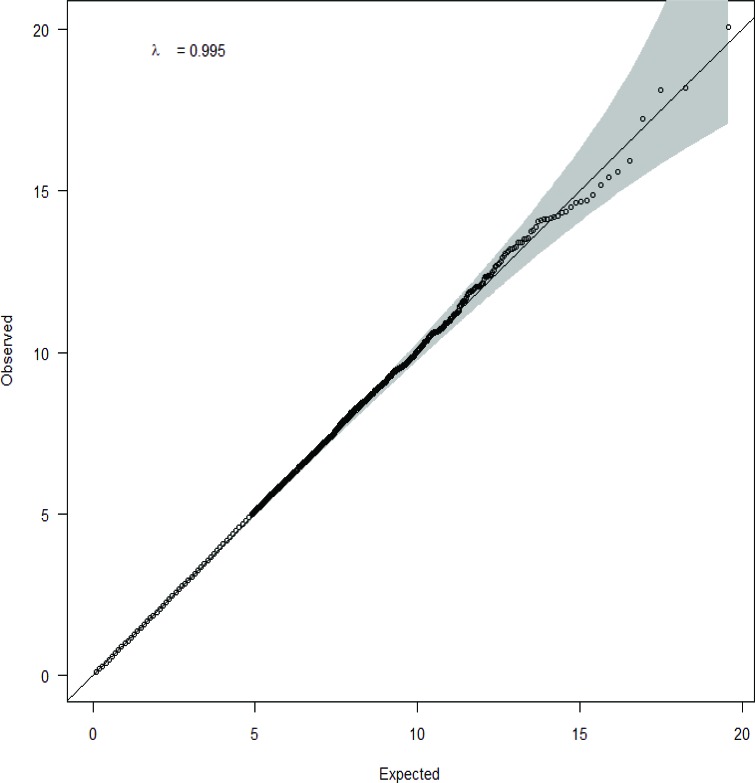
Q-Q plot of observed *versus* expected chi2 test statistics in Texas population

A total of 781 pathways, including those from KEGG (http://www.genome.jp/kegg/), Biocarta (http://cgap.nci.nih.gov/Pathways/BioCarta_Pathways), and Reactome (http://www.reactome.org/) databases were included in the GSEA of bladder cancer GWAS (Supplementary Figure 1). A quantile-quantile plot of observed *versus* expected chi2 test statistics showed no evidence for inflation in the Texas population (inflation factor 0.995)(Figure 1). We identified 85 possibly significant pathways associated with bladder cancer risk by Gen-Gen method alone (Supplementary Table 1), 44 significant pathways by Aligator (Supplementary Table 2), and 68 significant pathways by SNP Ratio Test (Supplementary Table 3). The results from the above three GSEAs (Gen-Gen, SNP Ratio Test, and Aligator) were consistent that we identified 18 novel pathways (*P* < 0.05) significantly associated with bladder cancer risk in all three GSEA methods (Table 1) from the Texas population. Top five pathways (*P* ≤ 0.001 in any of the three GSEA methods) among the 18 pathway included two cell cycle pathways involved in the G1/S transition (*P*GenGen: 0.001, *P*_Aligator_: 0.001, *P*_SRT_: 0.002), neural cell adhesion molecule (NCAM) pathway (*P*_GenGen_: < 0.001, *P*_Aligator_: 0.020, *P*SRT: 0.014), platelet-derived growth factor (PDGF) induced intracellular pathway (*P*_GenGen_: < 0.001, *P*_Aligator_: 0.026, *P*_SRT_: 0.006) and unfolded protein response pathway (*P*_GenGen_: 0.007, *P*Aligator: 0.001, *P*_SRT_: < 0.001) (Table 1). We grouped these five significant pathways into three categories based on functional similarity:

**Table 1:** Significant pathways from the three gene set enrichment analyses

	Gen-Gen		Aligator		SNP Ratio Test	
Pathway	P	Rank	P	Rank	P	Rank
**REACTOME_SIGNALING_BY_PDGF**	<0.001	1	0.0264	25	0.005994	5
**REACTOME_NCAM_SIGNALING_FOR_NEURITE_OUT_GROWTH**	<0.001	1	0.0198	21	0.013986	12
**BIOCARTA_RACCYCD_PATHWAY**	0.001	2	0.0012	2	0.001998	2
**BIOCARTA_SKP2E2F_PATHWAY**	0.001	2	0.0016	3	0.011988	10
BIOCARTA_NDKDYNAMIN_PATHWAY	0.003	4	0.007	8	0.08991	56
REACTOME_NCAM1_INTERACTIONS	0.005	6	0.009	11	0.025974	21
BIOCARTA_P27_PATHWAY	0.005	6	0.0044	6	0.021978	18
**REACTOME_UNFOLDED_PROTEIN_RESPONSE**	0.007	8	0.0008	1	0.000999	1
REACTOME_INACTIVATION_OF_APC_VIA_DIRECT_INHIBITION_OF_THE_APCOMPLEX	0.008	9	0.0368	30	0.002997	3
BIOCARTA_BAD_PATHWAY	0.009	10	0.0496	38	0.008991	7
REACTOME_CONVERSION_FROM_APC_CDC20_TO_APC_CDH1_IN_LATE_ANAPHASE	0.013	13	0.0484	37	0.016983	14
BIOCARTA_NFAT_PATHWAY	0.017	16	0.0036	4	0.013986	12
REACTOME_CTLA4_INHIBITORY_SIGNALING	0.018	17	0.0392	32	0.027972	22
REACTOME_PHOSPHORYLATION_OF_THE_APC	0.018	17	0.0376	31	0.010989	9
REACTOME_APCDC20_MEDIATED_DEGRADATION_OF_CYCLIN_B	0.019	18	0.0392	32	0.00999	8
KEGG_PROSTATE_CANCER	0.02	19	0.004	5	0.036963	29
REACTOME_SYNTHESIS_OF_BILE_ACIDS_AND_BILE_SALTS_VIA_24_HYDROXYCHOLESTEROL	0.025	22	0.007	8	0.048951	36
BIOCARTA_DC_PATHWAY	0.044	34	0.0138	17	0.005994	5

The pathways discussed in detail were in bold

### Cell cycle related pathways

**Figure 2:**
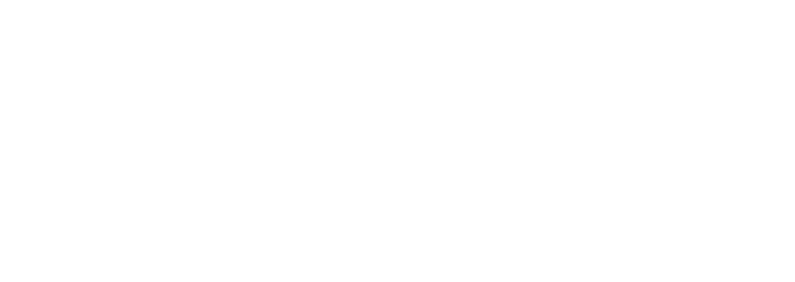
The imputation of gene regions of interest using 1000 genomes data (black dot) along with Texas Bladder Cancer GWAS data (gray dot) The SNPs indicated by triangles were those validated in NCI population. Top three gene regions in which top signals from imputation are in strong linkage disequilibrium (R^2^ > 0.7) with the SNPs validated in both Texas and NCI populations are displayed. **A.** *CCNE1*; **B.** *SKP1*; **C.** *CACNA1C*.

The two significant pathways, “BIOCARTA_ RACCYCD_PATHWAY” and “BIOCARTA_SKP2E2F_ PATHWAY”, were both related to cell cycle, specifically genes critical for G1 and S phase (Table 2 and Supplementary Figure 2A). In our GSEA, “BIOCARTA_ RACCYCD_PATHWAY” (http://www.biocarta.com/pathfiles/h_RacCycDPathway.asp) contains 300 SNPs from 26 genes, and “BIOCARTA_SKP2E2F_PATHWAY” (http://www.biocarta.com/pathfiles/h_skp2e2fPathway.asp) consists of 115 SNPs from 10 genes. Five genes (*E2F1*, *TFDP1*, *CDK2*, *RB1*, and *CCNE1*) overlapped in these two pathways (Table 2 and Supplementary Figure 2A). We also examined the individual SNP association for variants located in genes from the two pathways and then queried the significant SNPs (*P* < 0.05) in the NCI bladder cancer GWAS for validation (Table 3). Three SNPs from “BIOCARTA_RACCYCD_PATHWAY”, and four SNPs from “BIOCARTA_SKP2E2F_PATHWAY” were significant in the Texas population, NCI population, and pooled analysis. Three significant SNPs were in *CCNE1* gene: rs8102137 (*P*_Texas_ = 0.0003, *P*_NCI_ = 0.0005, *P*_Pooled_ = 1×10^−6^), rs997669 (*P*_Texas_ = 0.0031, *P*_NCI_ = 0.0019, *P*_Pooled_ = 3.6×10^−5^), and rs4804903 (*P*_Texas_ = 0.0458, *P*_NCI_ = 0.0019, *P*_Pooled_ = 0.001), which were not in linkage disequilibrium and were the overlapping SNPs co-existed in the two cell cycle pathways (Table 3). In addition, *SKP1* rs10491321 from “BIOCARTA_SKP2E2F_PATHWAY” remained significant after validation (*P*_Texas_ = 0.0206, *P*_NCI_ = 0.0035, *P*_Pooled_ = 0.0004) (Table 3). Imputation of *CCNE1* and *SKP1* region showed that the strongest signals in the region of interest are *CCNE1* rs60560217 (Chr19: 30288545) and *SKP1* rs7701836 (Chr5: 133539947) (Figure 2A and 2B), which are in strong linkage disequilibrium (R^2^ > 0.7) with *CCNE1* rs8102137 and *SKP1* rs10491321, a region already identified in previous GWAS [15].

**Table 2:** Genes contained in significant pathways

REACTOME_SIGNALING_BY_PDGF	HRAS*	PDGFB		PDGFA					BCAR1				STAT5A				STAT5B				PIK3CA				PDGFC			PDGFD		RAPGEF1
	PIK3CB	MAPK1*		NCK2					CRKL				NCK1				MAPK3*				COL1A2*				PDGFRA			PDGFRB		COL1A1*
64 genes	GRB2*	COL3A1*		COL2A1*					SRC*				STAT6				COL9A1*				COL9A2*				COL9A3*			KRAS*		COL6A6*
	SOS1*	COL6A3*		COL6A2*					COL6A1*				THBS1				THBS2				PIK3R1				THBS3			RASA1		PIK3R2
	THBS4	SPP1		COL4A4*					PLAT				COL4A3*				COL4A2*				COL4A1*				MAP2K1*			MAP2K2*		YWHAB*
	RAF1*	STAT1		COL5A2*					FURIN				PLG				STAT3				COL5A1*				COL4A5*			PTPN11		NRAS*
	PLCG1	COL29A1*	CRK					GRB7											
REACTOME_NCAM_SIGNALING_FOR_ NEURITE_OUT_GROWTH	NRTN	HRAS*		GDNF					ARTN				NCAM1				MAPK1*				MAPK3*				CNTN2			COL1A2*		COL1A1*
	PRNP	NCAN		SPTB					FGFR1				GRB2*				COL3A1*				CACNB1				CACNB2			COL2A1*		CACNB3
69 genes	CACNB4	ST8SIA2		SRC*					COL9A1*				COL9A2*				PTK2				COL9A3*				KRAS*			COL6A6*		SOS1*
	COL6A3*	COL6A2*		COL6A1*					AGRN				COL4A4*				COL4A3*				COL4A2*				COL4A1*			MAP2K1*		SPTBN5
	MAP2K2*	CREB1		CACNA1I					SPTBN4				PTPRA				YWHAB*				RAF1*				COL5A2*			CACNA1S		COL5A1*
	COL4A5*	RPS6KA5		PSPN					NRAS*				FYN				ST8SIA4				CACNA1G				SPTBN2			CACNA1H		SPTBN1
	GFRA1	COL29A1*			SPTA1					CACNA1F				GFRA4				CACNA1C				CACNA1D				GFRA2		SPTAN1		
BIOCARTA_RACCYCD_PATHWAY	E2F1*	HRAS		NFKBIA					NFKB1				AKT1				CCNE1*				RAC1				RHOA			PIK3CA		PAK1
	CHUK	PIK3R1		TFDP1*					RELA				RAF1				CDK6				RB1*				CDK4			CDK2*		MAPK1
26 genes	CCND1	CDKN1A				CDKN1B	IKBKG								MAPK3				IKBKB							
**BIOCARTA_SKP2E2F_PATHWAY** / 10 genes	CDC34	CCNA1				E2F1*			CUL1				TFDP1*				CDK2*				RB1*				CCNE1*			SKP2		SKP1
REACTOME_UNFOLDED_PROTEIN_RESPONSE	HERPUD1	MBTPS2				PDIA6			NFYA				EDEM1				DDIT3				ATF6				ATF4			ATF3		DNAJB9
19 genes	DNAJB11	XBP1				EIF2S1					ERN1	HSPA5				DNAJC3				MBTPS1				EIF2AK3					SERP1	

* The overlapping genes in “REACTOME_SIGNALING_BY_PDGF” and “REACTOME_NCAM_SIGNALING_FOR_NEURITE_OUT_GROWTH”, and “BIOCARTA_RACCYCD_PATHWAY” and “BIOCARTA_SKP2E2F_PATHWAY” were indicated by asterisk.

**Table 3:** Validated SNPs from significant pathways in independent populations

		Texas						NCI				Meta-Analysis					
		Case		Control				Case		Control		Case			Control		
SNP	Gene	MAF		MAF		*P*		MAF		MAF	*P*	MAF			MAF	*P*	
REACTOME_NCAM_SIGNALING_FOR_NEURITE_OUT_GROWTH																	
rs12416052	*CACNB2*	0.43	0.4		0.024		0.43		0.41		0.0488		0.43	0.41			0.00638
rs17611556	*CACNB2*	0.06	0.09		0.0148		0.06		0.07		0.0185		0.06	0.08			0.00194
rs1990240	*CACNA1C*	0.28	0.25		0.0163		0.28		0.26		0.0206		0.28	0.26			0.00229
rs2239062	*CACNA1C*	0.44	0.41		0.0194		0.46		0.44		0.0188		0.46	0.44			0.00353
rs2239117	*CACNA1C*	0.28	0.25		0.0097		0.28		0.27		0.0452		0.28	0.26			0.00491
rs2239118	*CACNA1C*	0.25	0.21		0.0024		0.25		0.23		0.0447		0.25	0.23			0.0024
rs3767499	*CACNA1S*	0.5	0.47		0.0325		0.49		0.47		0.0198		0.49	0.47			0.00224
rs418543*	*COL4A2*	0.37	0.33		0.0098		0.36		0.34		0.0049		0.36	0.34			0.00026
rs6011959*	*SRC*	0.31	0.28		0.032		0.28		0.26		0.0051		0.29	0.26			0.00019
rs7132154	*CACNA1C*	0.24	0.27		0.0313		0.23		0.25		0.0008		0.23	0.26			0.00011
rs7963955	*CACNA1C*	0.28	0.25		0.0149		0.28		0.26		0.0219		0.28	0.26			0.00231
REACTOME_SIGNALING_BY_PDGF																	
rs418543*	*COL4A2*	0.37	0.33		0.0098		0.36		0.34		0.0049		0.36	0.34			0.00026
rs6011959*	*SRC*	0.31	0.28		0.032		0.28		0.26		0.0051		0.29	0.26			0.00019
rs7040470	*RAPGEF1*	0.42	0.46		0.0089		0.43		0.46		0.0004		0.43	0.46			1.20E-05
BIOCARTA_RACCYCD_PATHWAY																	
rs4804903*	*CCNE1*	0.3	0.34		0.0019		0.33		0.34		0.0458		0.32	0.34			0.00106
rs8102137*	*CCNE1*	0.38	0.32		0.0006		0.35		0.33		0.0003		0.36	0.33			1.43E-06
rs997669*	*CCNE1*	0.44	0.39		0.0019		0.42		0.39		0.0031		0.42	0.39			3.61E-05
BIOCARTA_SKP2E2F_PATHWAY																	
rs10491321	*SKP1*	0.16	0.2		0.0035		0.19		0.2		0.0206		0.18	0.2			0.00045
rs4804903*	*CCNE1*	0.3	0.34		0.0019		0.33		0.34		0.0458		0.32	0.34			0.00106
rs8102137*	*CCNE1*	0.38	0.32		0.0006		0.35		0.33		0.0003		0.36	0.33			1.43E-06
rs997669*	*CCNE1*	0.44	0.39		0.0019		0.42		0.39		0.0031		0.42	0.39			3.61E-05
REACTOME_UNFOLDED_PROTEIN_RESPONSE																	
rs2518054	*HERPUD1*	0.13	0.11		0.0312		0.11		0.1		0.0193		0.12	0.1			0.00168

* “REACTOME_SIGNALING_BY_PDGF” and “REACTOME_NCAM_SIGNALING_FOR_NEURITE_OUT_GROWTH” had overlapping SNPs. The shared SNPs and Genes were indicated by asterisk. “BIOCARTA_RACCYCD_PATHWAY” and “BIOCARTA_SKP2E2F_PATHWAY” had overlapping SNPs. The shared SNPs and Genes were indicated by asterisk. MAF- minor allele frequency

There were two growth factor mediated intracellular signaling pathways significant in GSEA analysis. Neural cell adhesion molecule (NCAM) pathway, (“REACTOME_NCAM_SIGNALING_FOR_ NEURITE_OUT_GROWTH”: http://www.reactome.org/entitylevelview/PathwayBrowser.html#DB=gk_current&FOCUS_SPECIES_ID=48887&FOCUS_PATHWAY_ID=375165&ID=375165) was composed of 2,232 SNPs from 69 genes, and platelet-derived growth factor (PDGF) induced intracellular pathway (“REACTOME_SIGNALING_BY_PDGF”: http://www.reactome.org/entitylevelview/PathwayBrowser.html#DB=gk_current&FOCUS_SPECIES_ID=48887&FOCUS_PATHWAY_ID=186797&ID=186797) consisted of 1,423 SNPs from 64 genes (Table 1 and Table 2). There were 31 overlapping genes co-existed in these two pathways (Table 2). After validation in NCI population, eleven SNPs in 5 genes of the “REACTOME_NCAM_ SIGNALING_FOR_NEURITE_OUT_GROWTH” pathway, including *CACNB2* rs12416052, rs17611556; *CACNA1C* rs1990240, rs2239062, rs2239117, rs2239118, rs7132154, rs7963955; *CACNA1S* rs3767499; *COL4A2* rs418543; and *SRC* rs6011959, and three SNPs *COL4A2* rs418543, *SRC* rs6011959, and *RAPGEF1* rs7040470 from the “REACTOME_SIGNALING_BY_PDGF” pathway remained significant with P value less than 0.005 in pooled analysis (Table 3). Among them, *SRC* rs6011959 and *COL4A2* rs418543 were overlapping SNPs in two pathways, while *RAPGEF1* rs7040470 was the most significant SNP in this PDGF mediated pathway with pooled P value of 1.2 × 10^−5^. For all these genes, the most significant SNPs in imputation were labeled in Figure 2C. The top signals of *CACNA1C* are chr12:2659044:D and rs11062272 which are in linkage disequilibrium (R^2^ > 0.7) with the validated SNPs rs2239117 and rs2239118 (Figure 2C).

### Unfolded protein response

Unfolded protein response (UPR) pathway (REACTOME_UNFOLDED_PROTEIN_RESPONSE: http://www.reactome.org/entitylevelview/pathwayBrowser.html#DB=gk_current&FOCUS_SPECIES_ID=48887&FOCUS_PATHWAY_ID=381119&ID=381160&VID=3079930) with 257 SNPs from 19 genes showed strongest enrichment signal based on Aligator (*P* = 0.0008) and SNP Ratio Test (*P* = 0.0009), and ranked top 8 in Gen-Gen (*P* = 0.007) (Table 2 and Supplementary Figure 2D). However, after validation, only one SNP rs2518054 at *HERPUD1* was significant in the Texas population with *P* = 0.0193, in NCI population with *P* = 0.0312, and in pooled analysis with *P* = 0.0017 (Table 3). The top signal in imputation is not linked with the validated SNP in *HERPUD1* (data not shown).

## DISCUSSION

In this study, we pursued a pathway approach in the Texas bladder cancer GWAS data using three GSEA methods including Gen-Gen, Aligator, and SNP Ratio Test. We identified 18 promising pathways out of 781 predefined gene-sets, which were associated with bladder cancer risk according to our screening criteria. The top five significant pathways involved cell cycle control at G1 and S phase, NCAM and PDGF induced intracellular signaling, and unfolded protein response. From these top five pathways, 17 SNPs from *CCNE1*, *RAPGEF1*, *SKP1*, *HERPUD1*, *CACNB2*, *CACNA1C*, *CACNA1S*, *COL4A2*, *SRC*, *CACNA1C*, appear to be associated with bladder cancer risk and were subsequently observed in the NCI bladder cancer GWAS.

The pathways highlighted were two cell cycle related pathways “BIOCARTA_RACCYCD_PATHWAY”, and “BIOCARTA_SKP2E2F_PATHWAY” at G1 and S phase (Supplementary Figure 2A). The G1/S phase transition is the rate-limiting step in cell cycle. This process is sequentially and coordinately regulated by the formation of several Cyclin-Cyclin Dependent Kinase (CDK) complexes, for example, Cyclin-D -CDK4/6 complex for G1 progression, Cyclin-E -CDK2 complex for the G1-S transition, and Cyclin-A -CDK2 complex for S-phase progression [35, 36]. Disruption of these complexes leads to either cell cycle arrest or uncontrolled cell cycle proliferation. Somatic and germline alterations of this pathway had been found in bladder cancer and other tumors [37–40]. In particular, the expression of Cyclin E1 has been correlated with more advanced and invasive bladder cancer, as well as poor clinical outcomes [41]. In our GSEA, the most significant SNP after validation in NCI population is *CCNE1* rs8102137, which maps to the chromosome 19q12 at the 5′ flanking region of *CYCLIN E1* gene with P value of 1×10^−6^ (Table 3). This SNP has been reported in previous GWAS, which is reassuring for the analytical approach [15]. *CCNE1* rs8102137 is also linked with the top signal rs60560217 in our imputation results (Figure 2A). There were no functional implications for rs60560217. Rs997669, located at the intron 4 of *CCNE1*, was significantly associated with bladder cancer risk (pooled P value of 3.6×10^−5^) independent of the above two SNPs.

In the “REACTOME_NCAM_SIGNALING_FOR_ NEURITE_OUT_GROWTH” pathway (Supplementary Figure 2B), the neural cell adhesion molecule, NCAM, belongs to the immunoglobulin superfamily. The NCAM induced intracellular signaling not only functions in neuronal differentiation, synaptic plasticity, and regeneration, but is also involved in the regulation of growth factor signaling, and cytoskeletion, etc. In the “REACTOME_SIGNALING_BY_PDGF” pathway (Supplementary Figure 2C), the binding of Platelet-derived growth factors (PDGF) to its two tyrosine kinase receptor induces the receptor dimerization and autophosphorylation, and enables the activation of many downstream molecules, such as SRC, PI3K, CRK, STAT, SHP-2, NCK, GAP, SHC, GRB2, GRB7, and PLC-γ1. Therefore PDGF can elicit the crosstalk of many downstream pathways, for example RAS-RAF-MEK-MAPK and PI3K-AKT pathways, to influence diverse functions, such as cell growth and motility [42].

In growth factor mediated intracellular pathways (Supplementary Figure 2B and 2C), *RAPGEF1* rs7040470 was significantly associated with bladder cancer risk and the significance level reached 1.2×10^−5^ in the pooled analysis (Table 3). *RAPGEF1* maps to 9q34.13 and encodes the RAP guanine nucleotide exchange factor 1. RAPGEF1 regulates the RAS-CRK-RAP1 cellular signal transduction system which has shown abnormality in lung carcinogenesis [43, 44]. Rs7040470 is at the downstream near gene region of *RAPGEF1*.

UPR contributes to a critical decision point between homeostasis or apoptosis of cell (Supplementary Figure 2D). During the ER stress, UPR initially decreases protein translation and enhances the unfolded protein degradation response to enforce the cell to maintain a homeostastic status [45]. If unable to maintain homeostasis within a certain time, the cell will commit apoptosis. In the UPR pathway (Supplementary Figure 2D), rs2518054 of *HERPUD1*was the only significant SNP validated with pooled P value 0.002 (Table 3). *HERPUD1*, located at 16q13, is an endoplasmic reticulum (ER) resident protein which is up-regulated in response to ER stress [46]. Interestingly, variants in *HERPUD1* have been associated with the metabolic syndrome in GWAS [47–49].

The GSEA method is an attractive approach for identifying additional susceptibility signals but it does require both larger sample sizes and independent replication sets to conclusively establish novel loci. GSEA can detect evidence for subtle effects of multiple SNPs in the same gene set, though it does not dissect pleiotropy in a given region. In our GSEA, we not only validated one SNP at *CCNE1* from previous GWAS, but also highlighted several novel SNPs, genes, and pathways potentially involved in bladder cancer tumorigenesis (Tables 1-3). Since GSEA grouped millions of SNPs into hundreds of gene sets, the burden of multiple comparisons have been greatly reduced. In addition, incorporation of the biological knowledge into statistical analysis renders our finding more relevant to biological interpretation. The biggest challenge for GSEA is how to define a gene set as misclassification leads directly to a loss of power. Due to the complexity of cell biology, some of the gene sets will be inevitably redundant or overlapping. Thus, associations could be driven by significant genes that are overlapped in different pathways. Another major limitation of GSEA is that it can only assess SNPs in or near gene regions so non-genic variants are not considered. Furthermore, the analysis assumes SNPs having only local cis-effects, an assumption that may be limiting. Finally, we only validated the genes and SNPs but not the pathways associated with bladder cancer risk since the NCI data was derived from multiple GWAS genotyping panels (HumanHap 1M, HumanHap610-Quad, HumanHap610, and HumanHap550 equivalents) compared to the MD Anderson data of using a single beadchip (HumanHap610). Differences in gene coverage and the selection of tagSNPs for each gene region in the various Illumina GWAS panels precluded us from confirming the results at the pathway level.

In summary, we implemented three different GSEA methods as internal validation to identify the biological pathways consistently associated with bladder cancer risk and also validated our results in an independent NCI population, which may reduce false discovery in our findings. GSEA is a complementary tool to identify additional genetic contributions to the heritability of bladder cancer, and may also be applicable to clinical outcome studies [50] by incorporating the biological pathway information into GWAS analysis. Our findings may pinpoint potential pathway targets for cancer prevention and treatment and to improve the risk prediction model of bladder cancer. However, to pursue these strategies, further research are needed to validate, fine-map and conduct functional characterization to pinpoint the variants directly associated with bladder cancer risk.

## MATERIALS AND METHODS

### Study population for primary GWAS

Study population was derived from our previous published GWAS (14), which included a total of 969 Caucasian cases and 957 Caucasian controls. Cases were recruited from MD Anderson Cancer Center and Baylor College of Medicine between 1999 and 2007. They were newly diagnosed bladder cancer patients, histologically confirmed, and previously untreated (ICD codes 188.1- 188.9). There were no restrictions on age, sex, ethnicity, and cancer stage in case recruitment. Control subjects were recruited from Kelsey Seybold clinics and were frequency-matched to cases by age (±5 years), sex and ethnicity. We restricted our analysis to Caucasians, due to the small number of minority participants in our population. All epidemiology data were collected by trained interviewers after signing of the consent form by study participants. The study was approved by the institutional review boards of MD Anderson Cancer Center, Baylor College of Medicine, and Kelsey-Seybold Clinic. No inflation was found in the study population structure [14]. All leukocyte DNAs were genotyped by Illumina HumanHap610 chip. Quality control for genotyping has been described previously (14). Briefly, cases and controls were excluded from analysis if they had genotyping call rates less than 95%; were found on review not to be of European ancestry; or were found to be duplicated samples, not matched according to established criteria, or to have reported a sex that did not match with X chromosome heterozygosity. We also excluded samples that deviated by more than 4 standard deviation from other study subjects using similarity in genotypes implemented in PLINK [51]. We randomly selected 2% of the samples for duplicate genotyping. The concordance of SNP genotype calls was > 99% for duplicated samples. Among the 620,901 markers on HumanHap610 chip, we excluded those that were copy number variation markers, did not yield genotypes, variants with minor allele frequency (MAF) less than 0.01 or with call rate < 95%. We further removed SNPs that deviated from Hardy- Weinberg equilibrium in the controls at *P* < 0.0001. These procedures left 556,429 SNPs for the final analysis.

The validation population consists of the primary scan of the NCI bladder cancer GWAS (available on dbGaP), which includes five studies with 3,532 cases and 5,120 controls of European ancestry [15, 34]. These five studies are Spanish Bladder Cancer Study (SBCS), New England, Maine and Vermont Bladder Cancer Study (NEBCS-ME/VT), Alpha-Tocopherol, Beta-Carotene Cancer Prevention Study (ATBC), the American Cancer Society Cancer Prevention Study II Nutrition Cohort (CPS-II), and the Prostate, Lung, Colorectal and Ovarian Cancer Screening Trial (PLCO). The same ICD codes as Texas GWAS were used for patient selection.

### Pathway definition and annotation

The molecular signature database (http://www.broadinstitute.org/gsea/msigdb/) from Broad Institute was used to define gene sets/pathways, which were composed of positional gene sets, curated gene sets, motif gene sets, computational gene sets and GO gene sets. We downloaded the 880 canonical pathways for GSEA. To avoid the overly narrow and broad definition of a biological pathway, we confined the input pathway to contain 10-100 genes per pathway, resulting in 781 pathways in our GSEA analysis (Supplementary Figure. 1). Among these, 151 pathways were selected from KEGG (http://www.genome.jp/kegg/), 214 were from Biocarta (http://www.biocarta.com/), 377 were from Reactome (http://www.reactome.org/), and 39 were from other resources. Biocarta generally has the smallest pathway size in terms of the number of gene in each pathway, with a median gene number of 18 per pathway. In contrast, KEGG pathway has the largest size with a median gene number of 44 per pathway. The significant pathways selected by GSEA were input into the Ingenuity Pathway Analysis tools (http://www.ingenuity.com/index.html) for functional annotation.

### SNP-gene map

Gene information was downloaded from NCBI dbSNP build 36.3. SNP information was from the Illumina HumanHap610 chip and validated by USCS genome browser (http://genome.ucsc.edu/). SNPs were mapped to gene region and ±20KB upstream and downstream of gene boundaries to cover the gene coding region and most of the regulatory components.

### Statistical analysis

#### Data preparation

To assess the association between each SNP and disease status, we built a 2×2 contingency table by counting the number of times each possible allele appears in a case or control and allelic 1 degree of freedom (d.f.) test implemented in PLINK was performed similar to the primary GWAS analysis [14]. We conducted quantile-quantile plot analysis to assess the distribution of chi2 test statistics of all GWAS SNPs using the R installed package snpMatrix (http://www.bioconductor.org/packages/2.3/bioc/html/snpMatrix.html) and hexbin (http://cran.r-project.org/web/packages/hexbin/index.html). Deviation of observed data from expected results might indicate the possibility of population stratification, inadequacy of case-control matching, or differential genotyping in cases and controls. We randomly permuted the case-control status 1000 times in order to test the presence of differential genotyping. In each permutated data the same number of cases and controls was generated and an allelic 1 d.f. test statistic and P value was re-calculated for each SNP using the permuted case-control status. We applied three GSEA methods for comparison to determine which pathway(s) associated with bladder cancer were likely true findings, not derived by chance. For all methods, pathways with *P* values < 0.05 were considered significant.

### Pathway analysis

#### GenGen [28]

The statistic value of each gene was represented by the highest statistic value among all SNPs mapped to the gene and then sorted from largest to smallest (r_(1)_,…, r_(N)_) for all *N* genes in the GWAS dataset. For any given gene set S, composed of *N_H_* genes, a weighted Kolmogorov- Smirnov-like running-sum statistic was calculated that reflects the overrepresentation of genes within the set S at the top of entire ranked list of genes in the genome:

$$\mathrm{ES}(S)={\text{MAX}}_{1\leq j\leq N}\{\sum_{Gj*\in s,j*\leq j}\frac{|r_{\left(j*\right)}{|}^{\#}}{N_{R}}-\sum_{Gj*\in s,j*\leq j}\frac{1}{N-N_{H}}\}$$

where $N_{R}={\sum_{{\mathrm{Gj}}^{*}\in s}{|r}_{{(j}^{*})}|}^{p}$, p was the weight to genes with extreme statistic values. The enrichment score, ES(S), indicated the maximum deviation of the sum of the statistic values in gene set S from a set of randomly picked genes in the genome. Normalized enrichment score, $\mathrm{NES}=\frac{\mathrm{ES}(S)-\text{mean}[\mathrm{ES}(S,\Pi )]}{\mathrm{SD}[\mathrm{ES}(S,\Pi )]},$ was used which enabled the comparisons among different gene sets [27].

#### Aligator [30]

Aligator utilizes a predefined threshold P-value of 0.01 wherein significant SNPs were defined on the basis of less than the predefined threshold. If a gene had one or more than one significant SNP, the gene was considered significant. Assume the total number of significant gene was K in the overall data. The number of significant genes was counted for each gene set. To determine the statistical significance of the gene set, 5000 replicate gene lists were generated by randomly selecting SNPs from all available SNPs and adding the genes that encompass the SNP to the gene list until the size of the gene list reached K. The P-value for each gene set was evaluated by comparing the number of significant genes from the observed data to the number of significant genes from the 5000 replicate gene lists. To correct for multiple testing, the program randomly selected one replicate gene list as the observed data and sample 5000 gene lists with replacement from the 5000 replicate gene lists. P-values for each gene set were calculated as before, using permutation. This procedure was repeated 1000 times to determine whether there was a significant excess of significant gene sets.

#### SNP ratio test [29]

For a given pathway W, the SNP ratio r_w_ = the number of significant SNP in W / the number of SNPs in W. The empirical P value for a particular pathway, *P* = (s+1)/(N+1), where s is the number of simulated datasets that produce a ratio greater than or equal to the original ratio, and N is the total number of simulated datasets.

### Gene and SNP analysis

We selected SNPs identified in the pathway analyses above for validation in a second dataset from the already published NCI GWAS [15]. The P values of the distribution of genotypes of these SNPs between case and controls were assessed by allelic 1 degree of freedom (d.f.) test in both Texas and NCI populations. For SNPs to be considered “validated”, the differential distribution of the genotypes in cases *vs*. controls in the validation group is consistent with the discovery population (same direction of change), and also both their associations with bladder cancer risk are significant at *P* < 0.05. A meta-analysis of the Texas and NCI populations was also used to further support the findings. Imputation of the SNPs at gene region of interest for Texas bladder cancer GWAS data was conducted by Impute 2 software (The University of Oxford http://mathgen.stats.ox.ac.uk/impute/impute_v2.html) using 1000 genomes data (http://www.1000genomes.org/) as reference panel.

### SUPPLEMENTARY TABLES AND FIGURES


