# Supplementary material for: Pathway analysis of bladder cancer genome-wide association study identifies novel pathways involved in bladder cancer development
Source: Genes Cancer. 2016 Jul;7(7-8):229–39. doi: 10.18632/genesandcancer.113 (PMC5059113; doi:10.18632/genesandcancer.113)

Pathway analysis of bladder cancer genome-wide association study identifies novel pathways involved in bladder cancer development – Chen et al

Supplementary Table 1: 85 significant pathways from Gen-Gen (P≤0.05) and corresponding results in other methods.

	Gen-Gen		Aligator		SNP Ratio Test	
Pathway	*P*	*Rank*	*P*	*Rank*	*P*	*Rank*
**REACTOME_SIGNALING_BY_PDGF**	**<0.001**	**1**	**0.0264**	**25**	**0.005994**	**5**
**REACTOME_NCAM_SIGNALING_FOR_NEURITE_OUT_GROWTH**	**<0.001**	**1**	**0.0198**	**21**	**0.013986**	**12**
REACTOME_SIGNALING_BY_WNT	0.001	2	0.1978	148	0.223776	134
**BIOCARTA_RACCYCD_PATHWAY**	**0.001**	**2**	**0.0012**	**2**	**0.001998**	**2**
**BIOCARTA_SKP2E2F_PATHWAY**	**0.001**	**2**	**0.0016**	**3**	**0.011988**	**10**
KEGG_ADHERENS_JUNCTION	0.002	3	0.298	234	0.017982	15
KEGG_PATHOGENIC_ESCHERICHIA_COLI_INFECTION	0.002	3	0.3498	276	0.018981	16
KEGG_TGF_BETA_SIGNALING_PATHWAY	0.003	4	0.44001	317	0.104895	62
**BIOCARTA_NDKDYNAMIN_PATHWAY**	**0.003**	**4**	**0.007**	**8**	**0.08991**	**56**
KEGG_RNA_POLYMERASE	0.003	4	0.091	67	0.132867	80
REACTOME_REGULATION_OF_APC_ACTIVATORS_BETWEEN_G1_S_AND_EARLY_ANAPHASE	0.004	5	0.0414	34	0.126873	75
**REACTOME_NCAM1_INTERACTIONS**	**0.005**	**6**	**0.009**	**11**	**0.025974**	**21**
KEGG_PANCREATIC_CANCER	0.005	6	0.1064	81	0.02997	23
REACTOME_SIGNALING_BY_VEGF	0.005	6	0.49241	347	0.020979	17
BIOCARTA_RAS_PATHWAY	0.005	6	0.0056	7	0.156843	97
**BIOCARTA_P27_PATHWAY**	**0.005**	**6**	**0.0044**	**6**	**0.021978**	**18**
BIOCARTA_ARF_PATHWAY	0.006	7	0.0338	28	0.116883	71
REACTOME_SEMA3A_PLEXIN_REPULSION_SIGNALING_BY_INHIBITING_INTEGRIN_ADHESION	0.007	8	0.2266	176	0.141858	86
**REACTOME_UNFOLDED_PROTEIN_RESPONSE**	**0.007**	**8**	**0.0008**	**1**	**0.000999**	**1**
KEGG_OTHER_GLYCAN_DEGRADATION	0.007	8	0.0782	54	0.080919	52
REACTOME_DNA_REPLICATION_PRE_INITIATION	0.008	9	0.229	179	0.327672	191
REACTOME_LOSS_OF_NLP_FROM_MITOTIC_CENTROSOMES	0.008	9	0.0098	13	0.127872	76
**REACTOME_INACTIVATION_OF_APC_VIA_DIRECT_INHIBITION_OF_THE_APCOMPLEX**	**0.008**	**9**	**0.0368**	**30**	**0.002997**	**3**
**BIOCARTA_BAD_PATHWAY**	**0.009**	**10**	**0.0496**	**38**	**0.008991**	**7**
BIOCARTA_P35ALZHEIMERS_PATHWAY	0.011	11	0.069	49	0.02997	23
BIOCARTA_PLATELETAPP_PATHWAY	0.012	12	0.0536	41	0.046953	35

REACTOME_BETACATENIN_PHOSPHORYLATION_CASCADE	0.012	12	0.101	79	0.083916	53
REACTOME_ACTIVATION_OF_THE_PRE_REPLICATIVE_COMPLEX	0.013	13	0.1832	136	0.207792	126
BIOCARTA_ECM_PATHWAY	0.013	13	0.082	58	0.103896	61
REACTOME_CHOLESTEROL_BIOSYNTHESIS	0.013	13	0.0414	34	0.100899	60
**REACTOME_CONVERSION_FROM_APC_CDC20_TO_APC_CDH1_IN_LATE_ANAPHASE**	**0.013**	**13**	**0.0484**	**37**	**0.016983**	**14**
KEGG_PYRIMIDINE_METABOLISM	0.014	14	0.2648	210	0.278721	163
REACTOME_CENTROSOME_MATURATION	0.016	15	0.0166	19	0.165834	102
BIOCARTA_TCR_PATHWAY	0.016	15	0.0084	10	0.136863	84
KEGG_CARDIAC_MUSCLE_CONTRACTION	0.016	15	0.0078	9	0.060939	41
BIOCARTA_AKT_PATHWAY	0.016	15	0.0174	20	0.222777	133
**BIOCARTA_NFAT_PATHWAY**	**0.017**	**16**	**0.0036**	**4**	**0.013986**	**12**
REACTOME_TRAFFICKING_OF_AMPA_RECEPTORS	0.017	16	0.2598	206	0.063936	43
REACTOME_CDC20_PHOSPHO_APC_MEDIATED_DEGRADATION_OF_CYCLIN_A	0.017	16	0.119	91	0.176823	111
**REACTOME_CTLA4_INHIBITORY_SIGNALING**	**0.018**	**17**	**0.0392**	**32**	**0.027972**	**22**
REACTOME_AUTODEGRADATION_OF_CDH1_BY_CDH1_APC	0.018	17	0.0886	65	0.175824	110
BIOCARTA_CELLCYCLE_PATHWAY	0.018	17	0.1068	82	0.034965	27
**REACTOME_PHOSPHORYLATION_OF_THE_APC**	**0.018**	**17**	**0.0376**	**31**	**0.010989**	**9**
REACTOME_SCF_SKP2_MEDIATED_DEGRADATION_OF_P27_P21	0.019	18	0.0828	59	0.318681	183
REACTOME_ORC1_REMOVAL_FROM_CHROMATIN	0.019	18	0.3058	240	0.373626	216
**REACTOME_APCDC20_MEDIATED_DEGRADATION_OF_CYCLIN_B**	**0.019**	**18**	**0.0392**	**32**	**0.00999**	**8**
**KEGG_PROSTATE_CANCER**	**0.02**	**19**	**0.004**	**5**	**0.036963**	**29**
REACTOME_PECAM1_INTERACTIONS	0.02	19	0.0568	43	0.043956	34
REACTOME_PHASE_1_FUNCTIONALIZATION_OF_COMPOUNDS	0.021	20	0.39321	297	0.030969	24
REACTOME_TRAFFICKING_OF_GLUR2_CONTAINING_AMPA_RECEPTORS	0.021	20	0.2016	153	0.021978	18
REACTOME_TRANSFORMATION_OF_LANOSTEROL_TO_CHOLESTEROL	0.021	20	0.131	100	0.070929	46
BIOCARTA_G1_PATHWAY	0.024	21	0.1434	108	0.030969	24
BIOCARTA_MCM_PATHWAY	0.024	21	0.0836	60	0.040959	31
KEGG_COMPLEMENT_AND_COAGULATION_CASCADES	0.025	22	0.92095	529	0.107892	63
**REACTOME_SYNTHESIS_OF_BILE_ACIDS_AND_BILE_SALTS_VIA_24_HYDROXYCHOLESTEROL**	**0.025**	**22**	**0.007**	**8**	**0.048951**	**36**
REACTOME_MYD88_CASCADE	0.028	23	0.107	83	0.056943	39
BIOCARTA_CHREBP2_PATHWAY	0.029	24	0.83296	506	0.176823	111
REACTOME_CYCLIN_E_ASSOCIATED_EVENTS_DURING_G1_S_TRANSITION_	0.031	25	0.1136	88	0.306693	178
REACTOME_PLATELET_DEGRANULATION	0.032	26	0.0816	57	0.124875	74

REACTOME_G2_M_TRANSITION	0.032	26	0.0298	26	0.174825	109
REACTOME_MITOTIC_PROMETAPHASE	0.032	26	0.1146	89	0.011988	10
REACTOME_DOWN_STREAM_SIGNAL_TRANSDUCTION	0.032	26	0.0516	40	0.022977	19
REACTOME_FORMATION_OF_A_POOL_OF_FREE_40S_SUBUNITS	0.032	26	0.322	251	0.414585	244
BIOCARTA_WNT_PATHWAY	0.034	27	0.332	262	0.435564	253
BIOCARTA_INTRINSIC_PATHWAY	0.035	28	0.5578	379	0.310689	180
KEGG_LONG_TERM_DEPRESSION	0.036	29	0.17	123	0.011988	10
BIOCARTA_IGF1MTOR_PATHWAY	0.036	29	0.2034	155	0.53047	303
REACTOME_M_G1_TRANSITION	0.037	30	0.50601	357	0.263736	155
REACTOME_ACTIVATION_OF_CHAPERONES_BY_IRE1_ALPHA	0.037	30	0.073	51	0.012987	11
REACTOME_ZINC_INFLUX_INTO_CELLS_BY_THE_SLC39_GENES_FAMILY	0.04	31	0.224	175	0.124875	74
SA_REG_CASCADE_OF_CYCLIN_EXPR	0.042	32	0.1322	101	0.133866	81
REACTOME_PROSTANOID_HORMONES	0.043	33	0.0644	47	0.095904	58
REACTOME_STEROID_METABOLISM	0.044	34	0.0094	12	0.263736	155
**BIOCARTA_DC_PATHWAY**	**0.044**	**34**	**0.0138**	**17**	**0.005994**	**5**
BIOCARTA_SPRY_PATHWAY	0.045	35	0.0648	48	0.005994	5
KEGG_PORPHYRIN_AND_CHLOROPHYLL_METABOLISM	0.046	36	0.251	201	0.247752	150
REACTOME_GLUCOSE_AND_OTHER_SUGAR_SLC_TRANSPORTERS	0.046	36	0.1844	138	0.236763	144
BIOCARTA_UCALPAIN_PATHWAY	0.046	36	0.3418	270	0.36963	214
BIOCARTA_P53HYPOXIA_PATHWAY	0.046	36	0.021	23	0.10989	65
KEGG_RIBOSOME	0.046	36	0.64299	431	0.491509	282
REACTOME_SCF_BETA_TRCP_MEDIATED_DEGRADATION_OF_EMI1	0.048	37	0.3366	267	0.483517	276
REACTOME_PLATELET_ADHESION_TO_EXPOSED_COLLAGEN	0.048	37	0.59499	401	0.058941	40
REACTOME_RNA_POLYMERASE_III_TRANSCRIPTION	0.05	38	0.1432	107	0.271728	160
BIOCARTA_BCR_PATHWAY	0.05	38	0.1	76	0.002997	3
BIOCARTA_41BB_PATHWAY	0.05	38	0.3286	258	0.233766	141

The significant pathways in all three GSEA methods were in bold.

Supplementary Table 2. Significant pathways from Aligator (P≤0.05)

Pathway	*P*	*Rank*
REACTOME_UNFOLDED_PROTEIN_RESPONSE	0.0008	1
BIOCARTA_RACCYCD_PATHWAY	0.0012	2
BIOCARTA_SKP2E2F_PATHWAY	0.0016	3
BIOCARTA_NFAT_PATHWAY	0.0036	4
KEGG_PROSTATE_CANCER	0.004	5
BIOCARTA_P27_PATHWAY	0.0044	6
BIOCARTA_RAS_PATHWAY	0.0056	7
BIOCARTA_NDKDYNAMIN_PATHWAY	0.007	8
REACTOME_SYNTHESIS_OF_BILE_ACIDS_AND_BILE_SALTS_VIA_24_HYDROXYCHOLESTEROL	0.007	8
KEGG_CARDIAC_MUSCLE_CONTRACTION	0.0078	9
BIOCARTA_TCR_PATHWAY	0.0084	10
REACTOME_NCAM1_INTERACTIONS	0.009	11
REACTOME_STEROID_METABOLISM	0.0094	12
REACTOME_LOSS_OF_NLP_FROM_MITOTIC_CENTROSOMES	0.0098	13
REACTOME_INORGANIC_CATION_ANION_SLC_TRANSPORTERS	0.0114	14
REACTOME_CELL_SURFACE_INTERACTIONS_AT_THE_VASCULAR_WALL	0.0126	15
KEGG_PROGESTERONE_MEDIATED_OOCYTE_MATURATION	0.0132	16
BIOCARTA_DC_PATHWAY	0.0138	17
KEGG_TYPE_II_DIABETES_MELLITUS	0.0146	18
REACTOME_CENTROSOME_MATURATION	0.0166	19
BIOCARTA_AKT_PATHWAY	0.0174	20
BIOCARTA_TCAPOPTOSIS_PATHWAY	0.0174	20
REACTOME_NCAM_SIGNALING_FOR_NEURITE_OUT_GROWTH	0.0198	21
REACTOME_ACTIVATION_OF_BH3_ONLY_PROTEINS	0.0208	22
BIOCARTA_P53HYPOXIA_PATHWAY	0.021	23
REACTOME_AMINO_ACID_TRANSPORT_ACROSS_THE_PLASMA_MEMBRANE	0.023	24
REACTOME_SIGNALING_BY_PDGF	0.0264	25
REACTOME_G2_M_TRANSITION	0.0298	26
BIOCARTA_CSK_PATHWAY	0.0324	27
BIOCARTA_ARF_PATHWAY	0.0338	28

REACTOME_TCR_SIGNALING	0.0364	29
ST_G_ALPHA_S_PATHWAY	0.0364	29
REACTOME_INACTIVATION_OF_APC_VIA_DIRECT_INHIBITION_OF_THE_APCOMPLEX	0.0368	30
REACTOME_PHOSPHORYLATION_OF_THE_APC	0.0376	31
REACTOME_CTLA4_INHIBITORY_SIGNALING	0.0392	32
REACTOME_APCDC20_MEDIATED_DEGRADATION_OF_CYCLIN_B	0.0392	32
BIOCARTA_RAB_PATHWAY	0.0394	33
REACTOME_REGULATION_OF_APC_ACTIVATORS_BETWEEN_G1_S_AND_EARLY_ANAPHASE	0.0414	34
REACTOME_CHOLESTEROL_BIOSYNTHESIS	0.0414	34
REACTOME_PROTEOLYTIC_CLEAVAGE_OF_SNARE_COMPLEX_PROTEINS	0.0434	35
REACTOME_GAB1_SIGNALOSOME	0.0468	36
REACTOME_CONVERSION_FROM_APC_CDC20_TO_APC_CDH1_IN_LATE_ANAPHASE	0.0484	37
BIOCARTA_BAD_PATHWAY	0.0496	38
BIOCARTA_PTDINS_PATHWAY	0.0496	38

Supplementary Table 3. Significant pathways from SNP Ratio Test (P≤0.05)

Pathway	*P*	*Rank*
REACTOME_UNFOLDED_PROTEIN_RESPONSE	0.000999	1
KEGG_HOMOLOGOUS_RECOMBINATION	0.000999	1
BIOCARTA_RACCYCD_PATHWAY	0.001998	2
BIOCARTA_BCR_PATHWAY	0.002997	3
SIG_BCR_SIGNALING_PATHWAY	0.002997	3
REACTOME_INACTIVATION_OF_APC_VIA_DIRECT_INHIBITION_OF_THE_APCOMPLEX	0.002997	3
REACTOME_FORMATION_OF_ATP_BY_CHEMIOSMOTIC_COUPLING	0.003996	4
REACTOME_SIGNALING_BY_PDGF	0.005994	5
BIOCARTA_SPRY_PATHWAY	0.005994	5
BIOCARTA_DC_PATHWAY	0.005994	5
BIOCARTA_CERAMIDE_PATHWAY	0.005994	5
REACTOME_ACTIVATION_OF_BH3_ONLY_PROTEINS	0.007992	6
BIOCARTA_BAD_PATHWAY	0.008991	7
REACTOME_APCDC20_MEDIATED_DEGRADATION_OF_CYCLIN_B	0.00999	8
REACTOME_PHOSPHORYLATION_OF_THE_APC	0.010989	9
BIOCARTA_CALCINEURIN_PATHWAY	0.010989	9
REACTOME_MITOTIC_PROMETAPHASE	0.011988	10
KEGG_LONG_TERM_DEPRESSION	0.011988	10
BIOCARTA_SKP2E2F_PATHWAY	0.011988	10
REACTOME_ACTIVATION_OF_CHAPERONES_BY_IRE1_ALPHA	0.012987	11
BIOCARTA_GPCR_PATHWAY	0.012987	11
BIOCARTA_FCER1_PATHWAY	0.012987	11
REACTOME_NCAM_SIGNALING_FOR_NEURITE_OUT_GROWTH	0.013986	12
BIOCARTA_NFAT_PATHWAY	0.013986	12
BIOCARTA_EGF_PATHWAY	0.015984	13
REACTOME_DEPOLARIZATION_OF_THE_PRESYNAPTIC_TERMINAL_TRIGGERS_THE_OPENING_OF_CALCIUM_CHANNELS	0.016983	14
REACTOME_CONVERSION_FROM_APC_CDC20_TO_APC_CDH1_IN_LATE_ANAPHASE	0.016983	14
KEGG_ADHERENS_JUNCTION	0.017982	15
KEGG_PATHOGENIC_ESCHERICHIA_COLI_INFECTION	0.018981	16
REACTOME_SIGNALING_BY_VEGF	0.020979	17

BIOCARTA_P27_PATHWAY	0.021978	18
REACTOME_TRAFFICKING_OF_GLUR2_CONTAINING_AMPA_RECEPTORS	0.021978	18
BIOCARTA_VIP_PATHWAY	0.022977	19
REACTOME_DOWN_STREAM_SIGNAL_TRANSDUCTION	0.022977	19
REACTOME_ETHANOL_OXIDATION	0.024975	20
REACTOME_NCAM1_INTERACTIONS	0.025974	21
BIOCARTA_MYOSIN_PATHWAY	0.025974	21
KEGG_CYSTEINE_AND_METHIONINE_METABOLISM	0.027972	22
REACTOME_CTLA4_INHIBITORY_SIGNALING	0.027972	22
KEGG_PANCREATIC_CANCER	0.02997	23
BIOCARTA_P35ALZHEIMERS_PATHWAY	0.02997	23
BIOCARTA_G1_PATHWAY	0.030969	24
BIOCARTA_CELL2CELL_PATHWAY	0.030969	24
REACTOME_PHASE_1_FUNCTIONALIZATION_OF_COMPOUNDS	0.030969	24
REACTOME_REGULATION_OF_PYRUVATE_DEHYDROGENASE_COMPLEX	0.032967	25
REACTOME_SYNTHESIS_AND_INTERCONVERSION_OF_NUCLEOTIDE_DI_AND_TRIPHOSPHATES	0.033966	26
BIOCARTA_PYK2_PATHWAY	0.034965	27
BIOCARTA_CELLCYCLE_PATHWAY	0.034965	27
REACTOME_GRB2_EVENTS_IN_EGFR_SIGNALING	0.035964	28
REACTOME_ERKS_ARE_INACTIVATED	0.036963	29
KEGG_PROSTATE_CANCER	0.036963	29
KEGG_NON_SMALL_CELL_LUNG_CANCER	0.038961	30
KEGG_ONE_CARBON_POOL_BY_FOLATE	0.038961	30
BIOCARTA_MAL_PATHWAY	0.038961	30
BIOCARTA_MCM_PATHWAY	0.040959	31
REACTOME_PHASE_1_FUNCTIONALIZATION	0.040959	31
KEGG_GLYCOSPHINGOLIPID_BIOSYNTHESIS_GLOBO_SERIES	0.040959	31
REACTOME_INTRINSIC_PATHWAY_FOR_APOPTOSIS	0.041958	32
KEGG_GAP_JUNCTION	0.042957	33
REACTOME_CELL_SURFACE_INTERACTIONS_AT_THE_VASCULAR_WALL	0.042957	33
KEGG_ENDOMETRIAL_CANCER	0.043956	34
REACTOME_PECAM1_INTERACTIONS	0.043956	34
BIOCARTA_CARDIACEGF_PATHWAY	0.046953	35

REACTOME_PYRUVATE_METABOLISM	0.046953	35
BIOCARTA_PLATELETAPP_PATHWAY	0.046953	35
REACTOME_THROMBIN_SIGNALLING_THROUGH_PROTEINASE_ACTIVATED_RECEPTORS	0.046953	35
REACTOME_SYNTHESIS_OF_BILE_ACIDS_AND_BILE_SALTS_VIA_24_HYDROXYCHOLESTEROL	0.048951	36
BIOCARTA_HSP27_PATHWAY	0.048951	36

Supplementary Figure 1. Define the 781 pathways in gene set enrichment analysis.

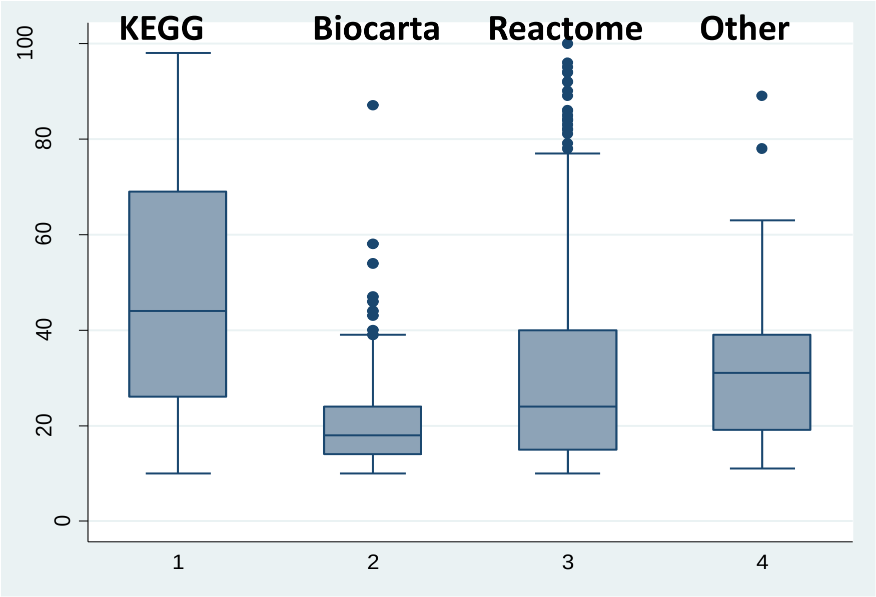

1. KEGG: 151 gene sets
   The number of gene per pathway: Range: 10-98; Median: 44; Mean: 47

2. Biocarta: 214 gene sets
   The number of gene per pathway: Range: 10-87; Median: 18; Mean: 21

3. Reactome: 377 gene sets
   The number of gene per pathway: Range: 10-100; Median: 24; Mean: 31

4. Other: 39 gene sets
   The number of gene per pathway: Range: 11-89; Median: 31; Mean: 33

Supplementary Figure 2A. The cell cycle pathway including Biocarta_RACCYCD_pathway and Biocarta_SKP2E2F pathway

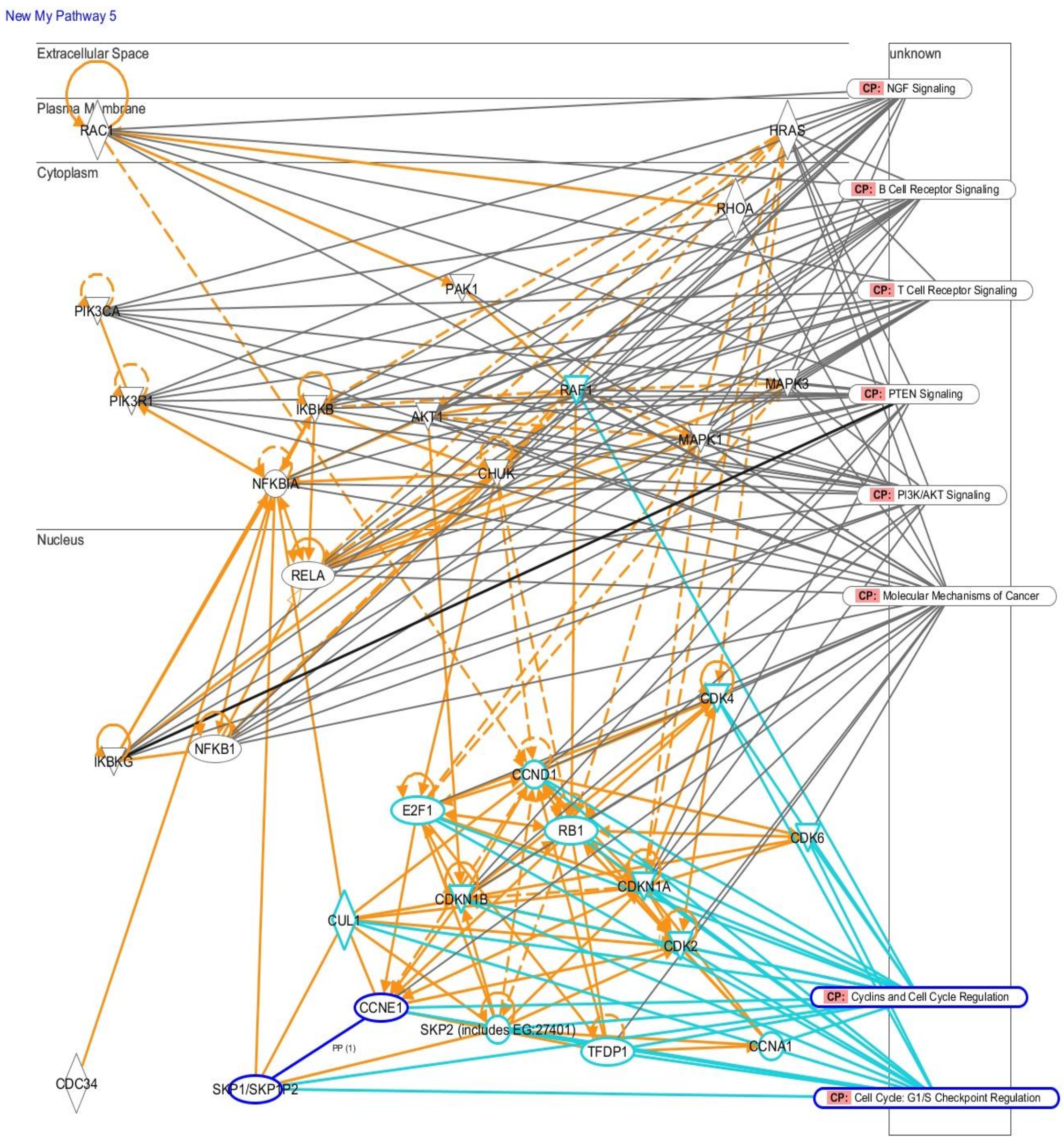

© 2000-2012 Ingenuity Systems, Inc. All rights reserved.

Supplementary Figure 2B. REACTOME_NCAM_SIGNALING_FOR_NEURITE_OUT_GROWTH

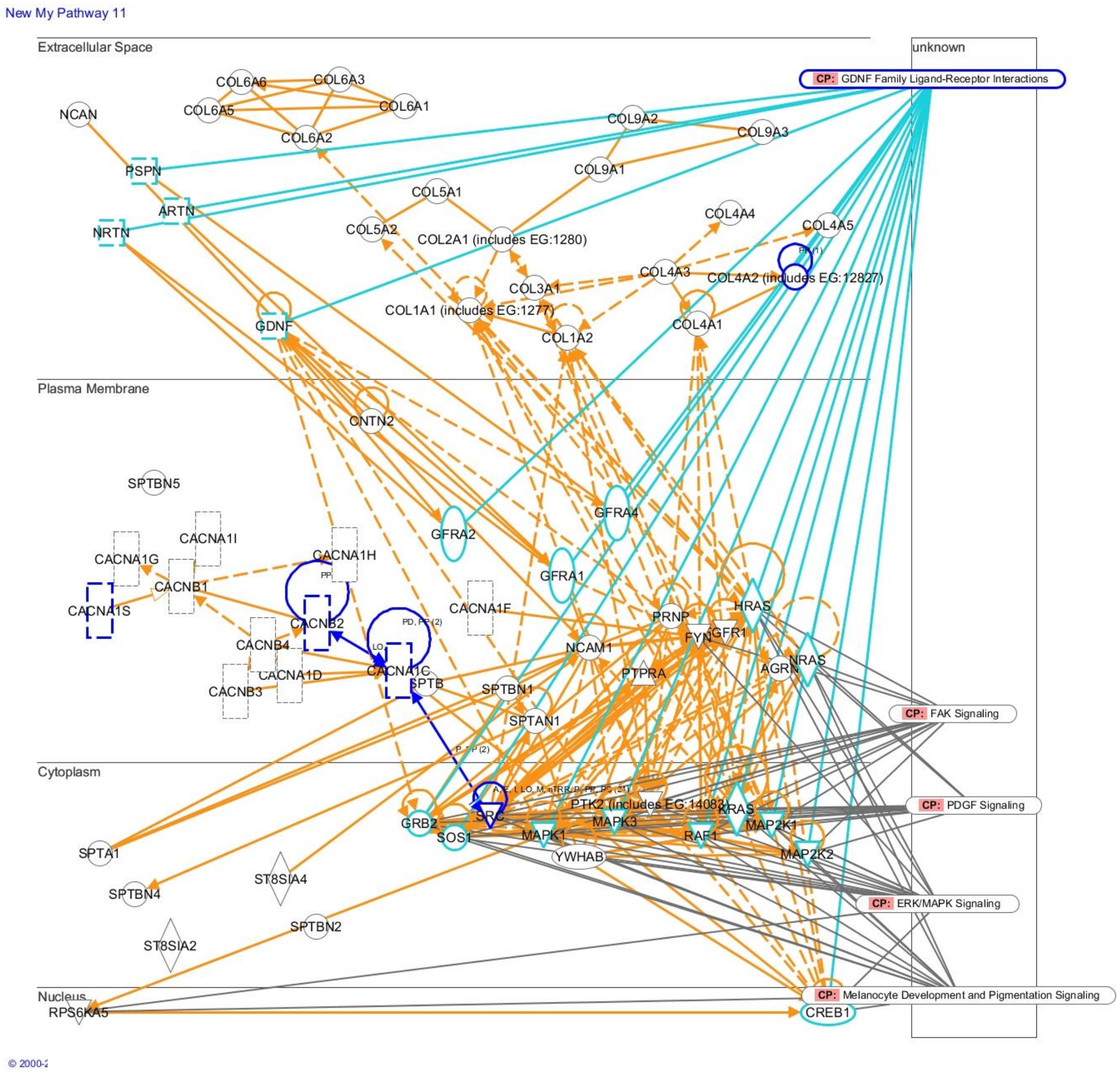

© 2000-2

Supplementary Figure 2C. REACTOME_SIGNALING_BY_PDGF

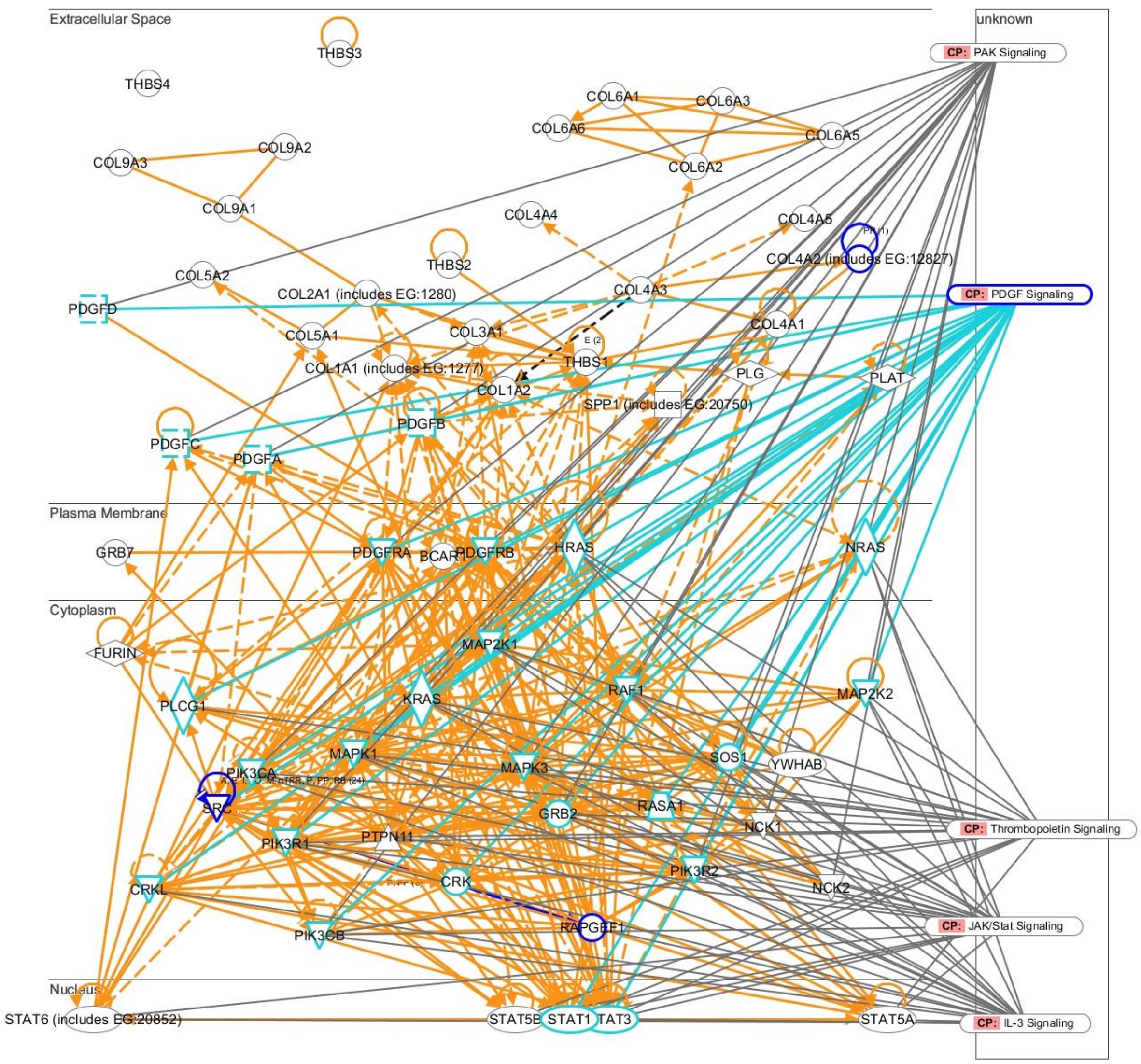

Supplementary Figure 2D. REACTOME_UNFOLDED_PROTEIN_RESPONSE

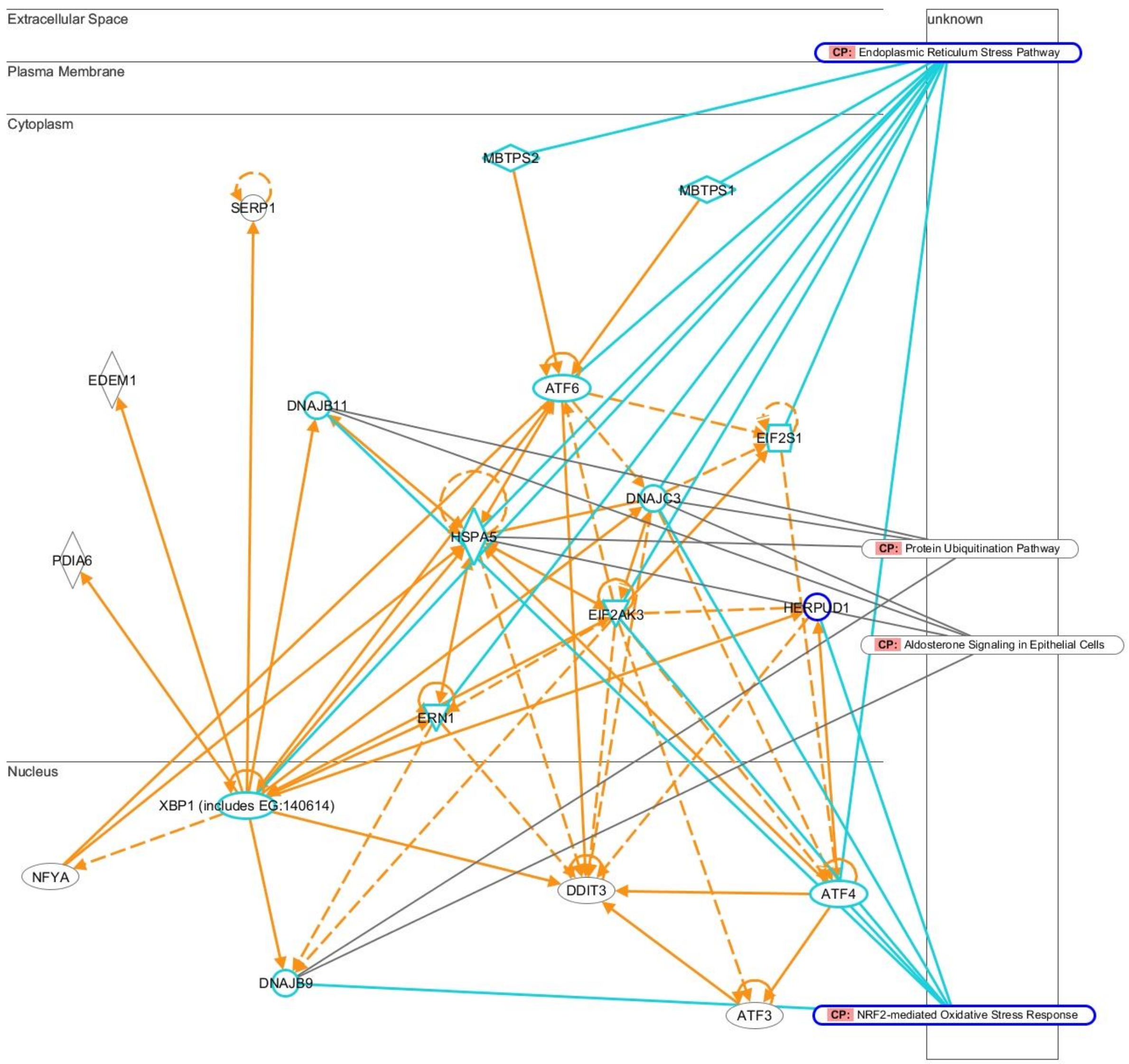

Supplement: Supplementary file 1 [file ganc-07-229-s001.pdf]
